# Development of a reverse genetics system for Sosuga virus allows rapid screening of antiviral compounds

**DOI:** 10.1371/journal.pntd.0006326

**Published:** 2018-03-09

**Authors:** Stephen R. Welch, Ayan K. Chakrabarti, Lisa Wiggleton Guerrero, Harley M. Jenks, Michael K. Lo, Stuart T. Nichol, Christina F. Spiropoulou, César G. Albariño

**Affiliations:** Viral Special Pathogens Branch, Division of High-Consequence Pathogens and Pathology, National Center for Emerging and Zoonotic Infectious Diseases, Centers for Disease Control and Prevention, Atlanta, GA, United States of America; Wistar Institute, UNITED STATES

## Abstract

Sosuga virus (SOSV) is a recently discovered zoonotic paramyxovirus isolated from a single human case in 2012; it has been ecologically and epidemiologically associated with transmission by the Egyptian rousette bat (*Rousettus aegyptiacus*). Bats have long been recognized as sources of novel zoonotic pathogens, including highly lethal paramyxoviruses like Nipah virus (NiV) and Hendra virus (HeV). The ability of SOSV to cause severe human disease supports the need for studies on SOSV pathogenesis to better understand the potential impact of this virus and to identify effective treatments. Here we describe a reverse genetics system for SOSV comprising a minigenome-based assay and a replication-competent infectious recombinant reporter SOSV that expresses the fluorescent protein ZsGreen1 in infected cells. First, we used the minigenome assay to rapidly screen for compounds inhibiting SOSV replication at biosafety level 2 (BSL-2). The antiviral activity of candidate compounds was then tested against authentic viral replication using the reporter SOSV at BSL-3. We identified several compounds with anti-SOSV activity, several of which also inhibit NiV and HeV. Alongside its utility in screening for potential SOSV therapeutics, the reverse genetics system described here is a powerful tool for analyzing mechanisms of SOSV pathogenesis, which will facilitate our understanding of how to combat the potential public health threats posed by emerging bat-borne paramyxoviruses.

## Author summary

Shortly after returning from a field trip to South Sudan and Uganda in 2012, a wildlife researcher presented to the emergency room with high fever and muscle and joint pain, that later progressed to severe disease. Sosuga virus (SOSV), a novel paramyxovirus, was identified as the etiological agent. Further epidemiological investigations identified bats as the probable reservoir of SOSV. Other related bat-borne paramyxoviruses, such as Nipah and Hendra viruses, have developed into major public health issues, raising concern that SOSV could represent an emerging threat. We developed a SOSV reverse genetics system to screen antiviral compounds and identify putative therapeutic interventions. Using this system, we generated a reporter virus capable of expressing a fluorescent protein in infected cells. By incorporating the reporter SOSV into a high-throughput screening assay, we identified several compounds that inhibit SOSV replication. In addition to antiviral screening, this rescue system can be used to investigate disease mechanisms, and to contribute to development of public health interventions for threats posed by emerging bat-transmitted paramyxoviruses.

## Introduction

The Paramxyoviridae (order *Mononegavirales*) represent a diverse family of viruses, many of which, such as measles virus, respiratory syncytial virus, and mumps virus, are significant human pathogens [1]. Within this family, bat-borne paramyxoviruses have previously been recognized as potential novel zoonotics with the ability to spill over into the human population and cause disease [2–4]. The 2 bat-transmitted paramyxoviruses with the greatest public health impact to date are Hendra (HeV) and Nipah (NiV) viruses, which together comprise the *Henipavirus* genus and are vectored by fruit bats of the *Pteropus* genus [5]. First recognized in 1994, HeV infections in both domestic horses and humans have occurred sporadically [6,7], while NiV infections in Bangladesh, India, and Malaysia have resulted in over 600 human infections to date [8].

Sosuga virus (SOSV) is a recently discovered zoonotic paramyxovirus [9]. It was first identified from a single clinical case in 2012, when a wildlife researcher who conducted a 6-week field expedition in South Sudan and Uganda presented with fever, generalized myalgia and arthralgia, neck stiffness, and a sore throat shortly upon returning to the USA [10]. The patient was hospitalized as symptoms worsened, including the development of a confluent maculopapular rash; after 14 days, all symptoms had sufficiently resolved to allow discharge. After several differential diagnoses were ruled out, sequence analysis revealed the presence of a novel rubula-like paramyxovirus in samples taken from the patient, from which the virus was later isolated. Further ecological and epidemiological investigations revealed Egyptian rousette bats (*Rousettus aegyptiacus*) as the likely reservoir for the virus, with this case representing a single spillover event into humans [11]. While only one case of SOSV infection has been confirmed to date, its detection in bat populations sampled over a 3-year period at multiple geographic locations in Uganda means that the possibility exists for future spillover events into the human population [11].

Reverse genetics systems allow for precise manipulation of viral genomes, and the ability to modify them to suit the required application. One such application is the generation of recombinant viruses expressing reporter proteins that facilitate indirect quantification of viral replication in infected cells. However, for pathogenic viruses such as SOSV, which require manipulation under high biocontainment levels, minigenome-based assays are also valuable surrogates that recapitulate viral replication and transcriptional processes without generating infectious particles. Here we describe a reverse genetics system for SOSV that can be used to rapidly screen for compounds that inhibit viral replication. An initial minigenome screen performed under biosafety level 2 (BSL-2) biocontainment was used to identify only those compounds with potential antiviral activity, which were then assessed for their ability to inhibit authentic viral replication processes using the reporter SOSV at BSL-3. As well as screening for antiviral compounds, these reverse genetics system will provide a powerful tool for analyzing mechanisms of SOSV pathogenesis in both in vitro and in vivo systems.

## Materials and methods

### Cells

Huh7 cells (ATCC, USA) were cultured in Dulbecco’s modified Eagle’s medium (DMEM) supplemented with 5% (v/v) fetal calf serum, non-essential amino acids, 100 U/mL penicillin, and 100 μg/mL streptomycin. BSR-T7/5 (generous gifts from Klaus Conzelmann, Germany), BHK-1 (ATCC, USA), and Vero-E6 cells (ATCC, USA) were cultured in DMEM supplemented with 10% (v/v) fetal calf serum, non-essential amino acids, 1 mM sodium pyruvate, 2 mM L-glutamine, 100 U/mL penicillin, and 100 μg/mL streptomycin.

### Reverse genetics: Minigenome assay and rescue of recombinant viruses

The SOSV minigenome segment was cloned into a Pol-I promoter-based plasmid (S1 Fig., Genbank #MG880223), and transfected into Huh7 cells in conjunction with Pol-II expression plasmids (pCAGGS) supplying SOSV polymerase (L), nucleoprotein (NP), and phosphoprotein (P) proteins in trans. The minigenome assay was performed in a 96-well plate on cells seeded at 2 × 10^4^ cells per well, with the following amounts of plasmids (150 ng total) transfected per well: 75 ng minigenome, 12.5 ng L, 50 ng NP, and 12.5 ng P. Minigenome activity was based on ZsGreen1 (ZsG) fluorescence levels determined at 72 h post transfection (hpt). To rescue recombinant virus, a T7 promoter-based plasmid containing full-length anti-genomic sense SOSV (S2 Fig.; Genbank #MG880224) was constructed. To rescue the reporter SOSV expressing ZsG, a modified genome was constructed in which the M open reading frame (ORF) was replaced with one encoding ZsG-P2A-M, leaving the M gene start and gene end intact (S2 Fig.; Genbank #MG880225). Virus rescue was performed in 6-well plates seeded with BSR-T7 cells (approximately 70% confluent) transfected with genome plasmid in conjunction with the SOSV-L, -NP, and -P expression plasmids (2 μg genome, 1 μg SOSV-L, 0.5 μg SOSV-NP, and 0.25 μg SOSV-P). At 96 hpt, cell culture supernatants were harvested, clarified by low-speed centrifugation, and used to infect BHK-21 cells. At 96 h post infection (hpi), cell culture supernatants were harvested, clarified, and titered by tissue culture infective dose 50 (TCID_50_) assays in Vero-E6 cells based on crystal violet visualization of cytopathic effects [12].

### Antiviral compound screening

All compounds were obtained from either Selleckchem (Houston, TX, USA) or Sigma-Aldrich (Saint Louis, MO, USA) with the exception of T-705 (BOC Sciences, Shirley, NY, USA) and 09167 (Vitas-M Laboratory, Champaign, IL, USA). For both screening assays, Huh7 cells were seeded in a 96-well plate at 2 × 10^4^ cells per well 16–20 h prior to treatment with DMSO-diluted compounds (final DMSO concentration 0.5%). For the minigenome screening assay, cells were transfected with the required plasmids using TransIT- LT-1 (Mirus Bio, Madison, WI, USA) 1 h post treatment. Total ZsG fluorescence was determined 72 hpt using a microplate reader (H1MD, BioTek Synergy, Winooski, VT, USA), with levels recorded at a height of 6 mm with 75 gain/sensitivity. ZsG levels were normalized to levels in mock-treated cells. For the reporter and wild-type screening assays, cells were infected 1 h post treatment with either rSOSV/ZsG or rSOSV at MOI 0.2. At 48 hpi, supernatants were collected (rSOSV-infected cells) for titer determination, or total ZsG fluorescence in the infected cell monolayer determined (rSOSV/ZsG-infected cells) using a H1MD plate reader (height 6 mm; 100 gain/sensitivity). For both assays, cell viability was determined by measuring ATP content in parallel with compound-treated, mock-transfected/infected cells using CellTiter-Glo 2.0 (Promega, Madison, WI, USA). All experiments were performed in quadruplicate and repeated at least 3 times.

### Microscopy and high-content imaging

ZsG fluorescence in infected cells was imaged directly using an EVOS digital inverted microscope (Thermo-Fisher, Waltham, MA, USA). For immunofluorescence, cells were fixed in 10% formalin for 30 min, permeabilized in PBS containing 0.1% Triton-X100 (v/v) for 10 min, and blocked with PBS 5% BSA (v/v) for 30 min. Primary antibody was rabbit α-SOSV NP (Genscript, Waltham, MA, USA), visualized with an AlexaFluor 488-labeled secondary antibody (Thermo-Fisher, #A11034). Quantification of total rSOSV protein production in Huh7 cells was performed on infected cells labeled as described above, and further stained with Nuc-Blue and Cell-Mask Red (Thermo-Fisher). Images were collected on the Operetta high-content imaging system (PerkinElmer, Waltham, MA, USA) using a 20× objective, with 16 fields per well analyzed using the Harmony software package (PerkinElmer). Total 488 fluorescence per field was determined, averaged for the well, and then normalized to values from control wells containing rSOSV-infected, mock-treated cells.

### Data analysis

ZsG fluorescence values for compound-treated, rSOSV/ZsG-infected cells were normalized to those in mock-treated (DMSO only) cells, and used to fit a 4-parameter equation to semilog plots of the concentration-response data. From this, the compound concentrations inhibiting 50% of the ZsG expression (50% effective concentration, EC_50_) were interpolated. Cell viability was similarly calculated in compound-treated, mock-infected cells to determine the 50% cell cytotoxicity concentration (CC_50_) of each compound. The selectivity index (SI) was calculated by dividing the CC_50_ by the EC_50_. Data analysis was performed using GraphPad Prism v7. Suitability for high-throughput screening was determined using the Z prime (Z′) score, a measure of statistical effect size, with values between 0.5 and 1.0 considered acceptable [13].

### Accession numbers

SOSV Minigenome plasmid; pSOSV MG; Genbank # MG880223

Rescue plasmid for rSOSV; pSOSV FL; Genbank #MG880224

Rescue plasmid for rSOSV/ZsG; pSOSV/ZsG FL, Genbank #MG880225

## Results

### Screening of antiviral compounds using a SOSV minigenome assay

**Fig 1:**
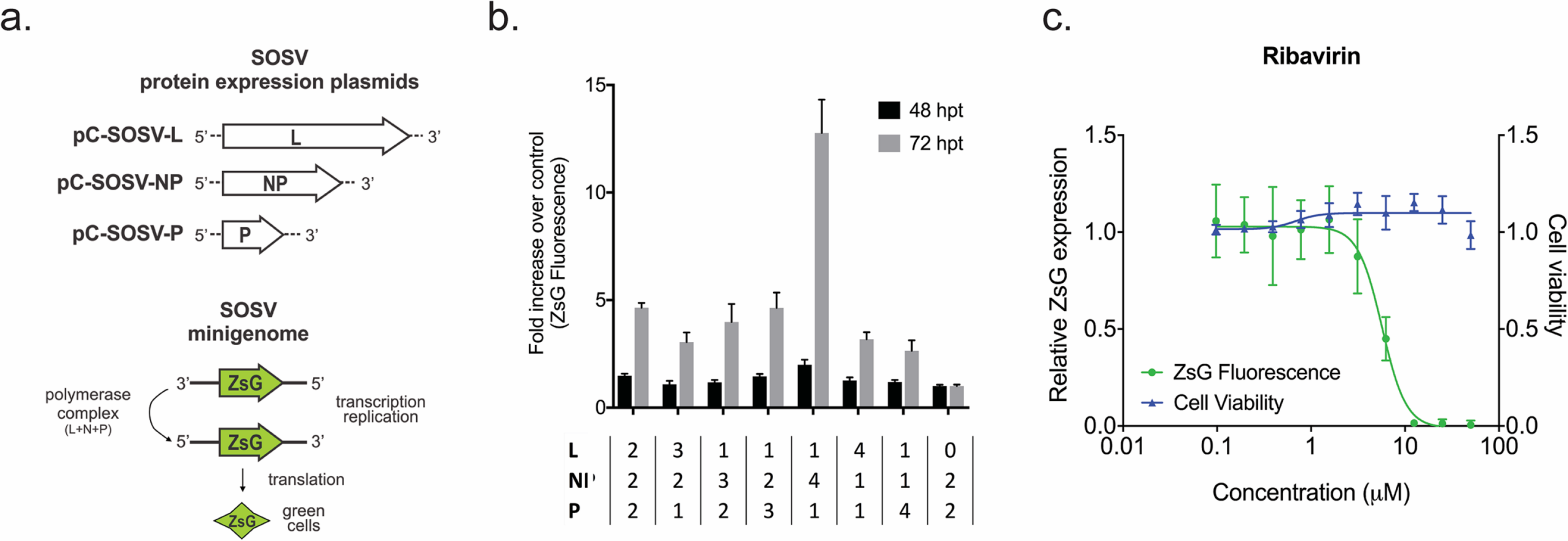
Design and optimization of the Sosuga virus minigenome screening assay. **a.** Genome schematic representing the design of the Sosuga virus (SOSV) minigenome segment. The minigenome contains the full-length SOSV 3′ and 5′ leader and trailer sequences, along with the gene start sequence for nucleoprotein (NP) and the gene end sequence for the viral polymerase (L), with the parental coding and intergenic regions replaced by the coding sequence of ZsGreen1 (ZsG). Transfected into cells in conjunction with plasmids expressing SOSV L, NP, and phosphoprotein (P), this minigenome allows for expression of quantifiable ZsG. **b.** The minigenome assay was optimized for Huh7 cells in a 96-well plate format using different ratios of the plasmids expressing L, NP, and P with a constant amount of minigenome plasmid. Cells were transfected with 75 ng minigenome plasmid and 75 ng total of plasmids expressing L, NP, and P, with ratios of each stated underneath the bars. Relative ZsG fluorescence over control reactions with no L was calculated at 48 and 72 h post transfection (hpt). **c.** Dose-response curve for the optimized SOSV minigenome assay against ribavirin. Cells were treated with a serial 2-fold dilution of ribavirin 1 hpt with the minigenome plasmids, and ZsG fluorescence was measured at 72 hpt. ZsG fluorescence (green) was normalized to mock-treated cells (DMSO only). Cell viability (blue) was determined concurrently by measuring ATP content, with values normalized to mock-transfected cells. Each point represents the mean of quadruplicate wells, with error bars showing standard deviation; graph is representative of 3 independent experiments.

We constructed a SOSV minigenome segment containing the parental SOSV 3′ and 5′ leader and trailer sequences, along with the gene start sequence for NP and the gene end sequence for L. All internal coding sequences and intergenic regions were replaced with the coding sequence for ZsGreen1 (ZsG), a green fluorescent protein (Fig 1A). Protein expression plasmids under control of the Pol-II promoter (pCAGGS) allowed in trans expression of SOSV NP, L, and P, the minimum requirement to initiate paramyxovirus replication and transcription [1]. The minigenome segment was cloned into a Pol-I expression plasmid in the negative orientation, meaning it has to be replicated first by the NP + L + P replication complex before transcription of ZsG mRNA can occur.

Next, the SOSV minigenome screen was optimized to achieve maximum reporter protein expression with the greatest signal-to-noise ratio in a 96-well plate format using Huh7 cells. Huh7 cells have previously proven useful for evaluating nucleoside analog activity against other viruses, and are highly permissive to SOSV infection [14–17]. Alongside a constant amount of minigenome plasmid, differing ratios of pCAGGS-L, -NP, and -P were transfected, and ZsG fluorescence was determined at both 48 and 72 hpt (Fig 1B). We determined the optimal ratio of plasmids for the assay to be 6:1:4:1 of genome, pCAGGS-L, -NP, and–P respectively, with ZsG fluorescence read at 72 hpt. To determine whether the optimized minigenome assay was suitable for screening antiviral compounds, we used the broad-spectrum antiviral ribavirin. Using 50 μM ribavirin as the positive control and DMSO as the vehicle-only control, the Z′ score of the assay was 0.58. Dose-dependent reduction of ZsG fluorescence was also observed in the SOSV minigenome assay using a serial 2-fold dilution of ribavirin, giving an EC_50_ of 5.91 ± 0.85 μM with no cytotoxicity (CC_50_ > 50 μM; Fig 1C). These data indicated that the assay was robust, and suitable for screening anti-SOSV compounds at BSL-2.

**Fig 2:**
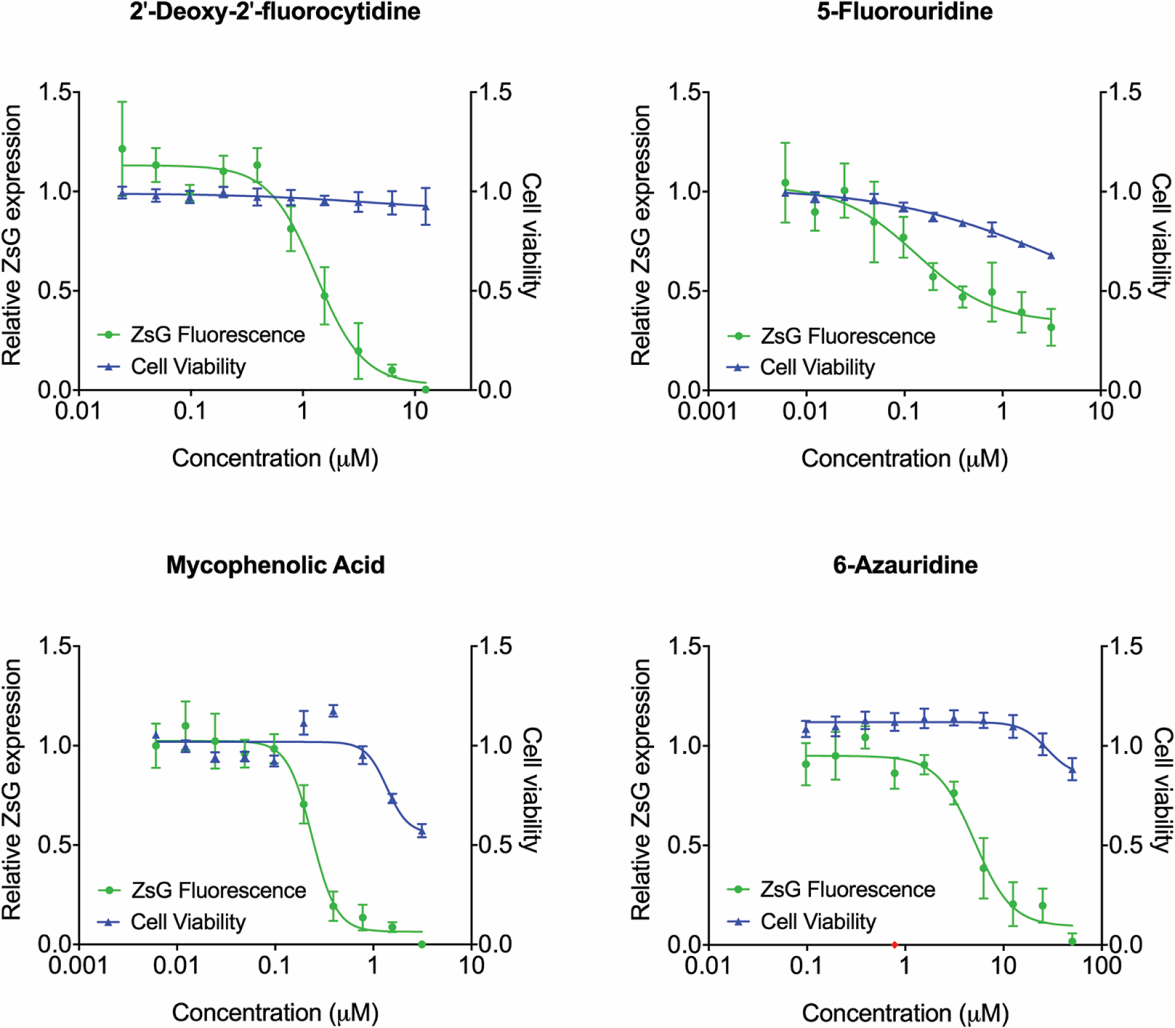
Dose-response curves for selected compounds using the minigenome assay. Compounds selected during the initial screen as potential SOSV antivirals were assessed by concentration-response curves using serial 2-fold dilutions of each compound. ZsG fluorescence (green) at 48 hpt was normalized to levels in mock-treated cells (DMSO only). Cell viability (blue) was determined concurrently by measuring ATP content, with values normalized to those in mock-transfected cells. Graphs are representative of at least 3 independent experiments. Each point represents the mean of quadruplicate wells, with errors bars indicating the standard deviation.

As minigenome assays can be used as surrogates for measuring viral replication and transcription, we evaluated a panel of 40 compounds predicted to inhibit these processes. These were a collection of nucleoside analogs supplemented with other select compounds reported to inhibit viral replication. Compounds were initially screened for activity at concentrations of 5000, 500, and 50 nM, with cell viability determined concurrently by assessing ATP content (S1 Table). Compounds inhibiting the minigenome by >50% whilst retaining >80% cell viability were then chosen for further analysis; these were 2′-deoxy-2′-fluorocytidine (2′-dFC), 5-fluorouridine, mycophenolic acid (MPA), and 6-azauridine. Dose-response analysis of these compounds demonstrated that both 2′-dFC and 6-azauridine inhibited minigenome activity in the absence of cell toxicity, with EC_50_ values of 1.31 ± 0.15 μM and 5.17 ± 1.01 μM, respectively (Fig 2). While 5-fluorouridine was not cytotoxic at the concentrations evaluated, it did not completely inhibit minigenome signal even at the highest concentrations evaluated, and was therefore dropped at this point. MPA inhibited minigenome activity at sub-micromolar concentrations, but was cytotoxic at higher concentrations. However, with an SI of 7, it was incorporated into the secondary virus screen to determine if a larger therapeutic window became apparent in the context of authentic viral replication.

### Design and rescue of a reporter SOSV expressing a fluorescent protein

**Fig 3:**
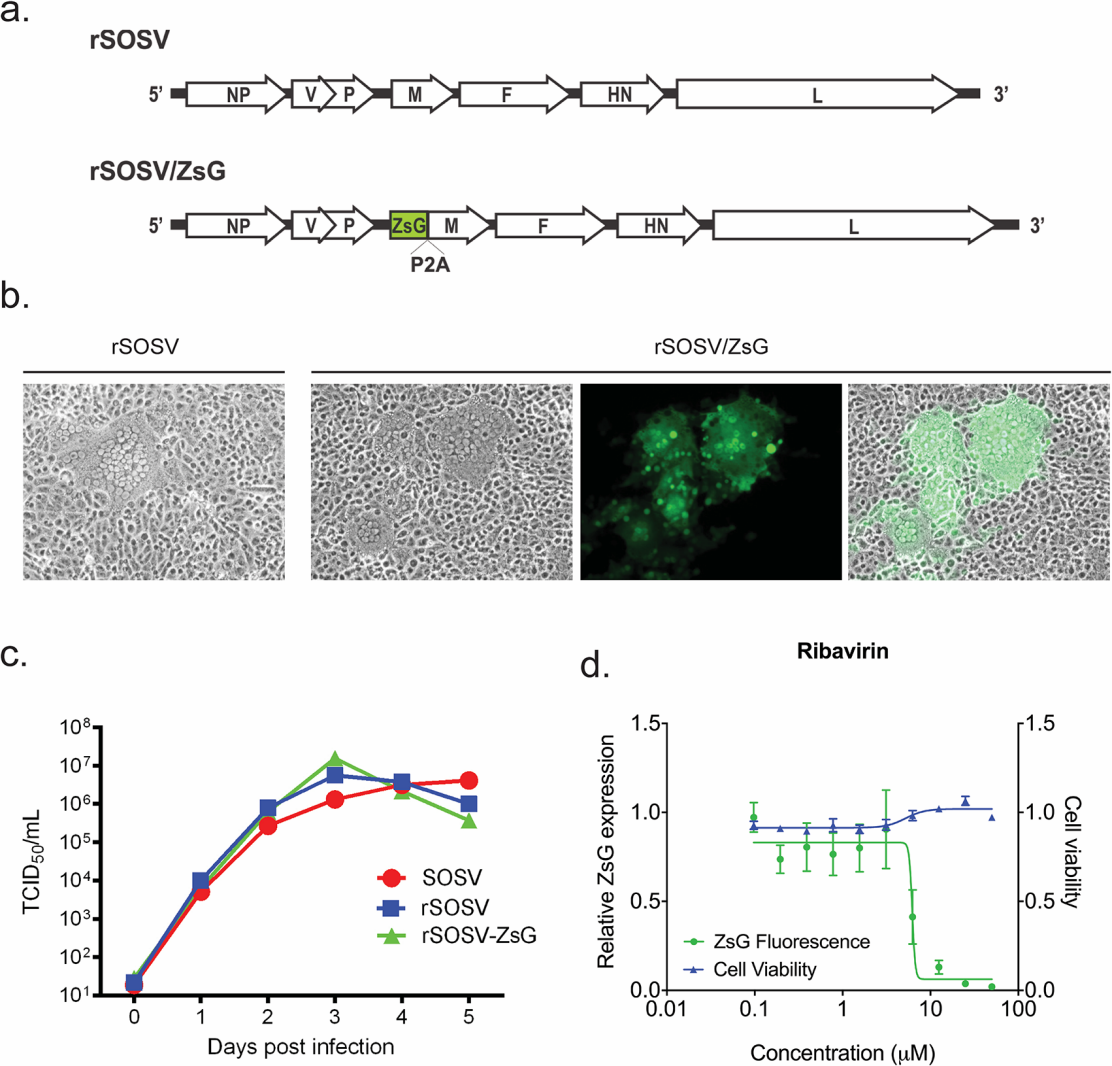
Optimization of an antiviral screening assay using a recombinant SOSV expressing ZsG. **a.** Schematic representation of the wild-type recombinant SOSV (rSOSV), and the reporter SOSV expressing ZsG (rSOSV/ZsG). The ZsG coding sequence was inserted immediately 5′ of the M coding sequence (antigenomic sense), separated from the viral protein by the P2A sequence from porcine teschovirus 1. This self-cleaving amino acid motif allows expression of both ZsG and M from a single mRNA generated by the parental SOSV M promoter and terminator sequences. **b.** Vero-E6 cells infected with either rSOSV or rSOSV/ZsG at 72 hpi showing formation of syncytia. In rSOSV/ZsG-infected cell monolayers, syncytia were associated with extensive ZsG expression (images taken at 4 × magnification). **c.** Growth curves for wild-type SOSV, rSOSV, and rSOSV/ZsG were performed in Vero-E6 cells infected at MOI 0.1. Titers (TCID_50_) were determined at 24, 48, 72, 96, and 120 hpi. **d.** The optimized rSOSV/ZsG antiviral screening assay was validated using ribavirin. Huh7 cells were treated with a serial 2-fold dilution of ribavirin 1 h prior to infection at MOI 0.2. ZsG fluorescence (green) was measured at 72 hpi and normalized to levels in mock-treated cells (DMSO only). Cell viability (blue) was determined concurrently by measuring ATP content, with values normalized to mock-infected cells. Each point represents the mean of quadruplicate wells, with error bars showing standard deviation; graph representative of 3 independent experiments.

The single-stranded, negative-sense RNA genome of SOSV encodes 6 genes to express 7 proteins, with 1 protein generated by V/P gene mRNA editing. The intergenic regions of SOSV contain transcriptional promoter and terminator sequences. We cloned the full-length SOSV genome into an expression plasmid under control of the T7 polymerase promoter. When transfected into cells and supplied with SOSV P, NP, L, and T7 polymerase in trans, this system successfully generated infectious recombinant SOSV (rSOSV). After optimizing the rSOSV rescue system, we designed another recombinant SOSV genome capable of expressing a fluorescent reporter protein in infected cells. Previous approaches to develop recombinant paramyxoviruses expressing reporter genes have included the reporter as either a separate open reading frame (ORF), or incorporated it within a parental ORF [18–22]. We employed the latter approach, and generated a SOSV genome in which the ZsG ORF was inserted upstream of the M ORF, fused to the viral protein via the self-cleaving P2A motif taken from porcine teschovirus 1 [23]. The authentic SOSV M gene start and gene end sequences therefore transcribe a single mRNA encoding the ORF for both M and ZsG, which, upon translation, results in the expression of 2 separate proteins via the P2A ribosomal skipping motif. Incorporation of this recombinant SOSV genome into the previously described SOSV rescue system (Fig 3A) resulted in the successful rescue of the reporter SOSV (rSOSV/ZsG).

Infecting Vero-E6 cells with rSOSV/ZsG at MOI 0.1 resulted in extensive syncytia formation in the cell monolayer similar to infection with rSOSV, with strong ZsG fluorescence detectable in these infected cells (Fig 3B). To compare growth kinetics of the recombinant viruses, Vero-E6 cells were infected with rSOSV, rSOSV/ZsG, or wild-type SOSV at MOI 0.1, with titers determined by TCID_50_ at 24, 48, 72, 96, and 120 hpi. All 3 viruses grew to similar titers over the 120 h time course, with no attenuation apparent in either of the recombinant viruses compared to the wild-type (Fig 3C). This indicated that incorporation of the ZsG ORF into the SOSV genome did not negatively impact the virus phenotype. To assess whether rSOSV/ZsG could be used to confirm antiviral activity of a compound by measuring the reduction in ZsG fluorescence in treated cells, we optimized a screening assay in Huh7 cells using 50 μM ribavirin as the positive control; the resultant Z′ score was 0.74, indicating a robust assay. A concentration-response curve using a 2-fold serial dilution of ribavirin demonstrated dose-dependent reduction in ZsG fluorescence in rSOSV/ZsG-infected cells, giving an EC_50_ of 6.10 ± 0.09 μM with no cytotoxicity (CC_50_ > 50 μM; Fig 3D).

### Screening of candidate antiviral compounds with recombinant viruses

**Fig 4:**
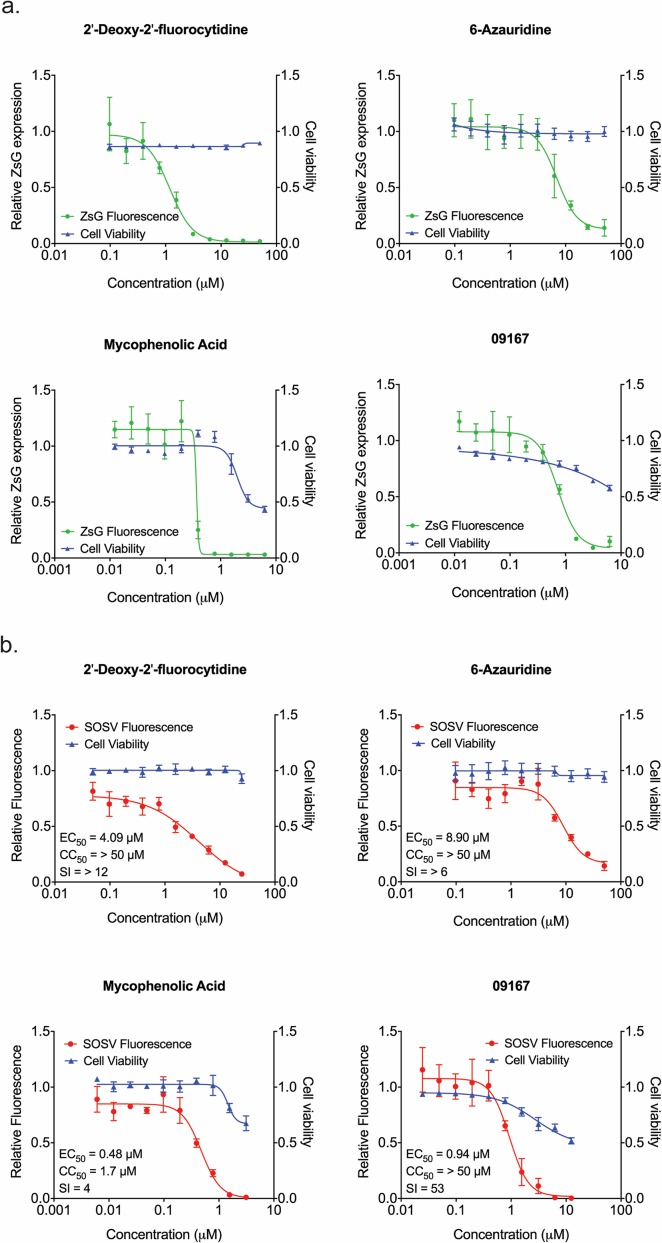
Using SOSV/ZsG as a tool to screen antiviral compounds. **a.** Representative concentration-response curves of Huh7 cells treated with 2-fold serial dilutions of compound 1 h prior to infection with rSOSV/ZsG at MOI 0.2. ZsG fluorescence (green) was measured at 48 hpi and normalized to mock-treated cells. Cell viability was assessed concurrently by determining ATP content (blue), with values normalized to mock-infected cells. Each point represents the mean of quadruplicate wells, with error bars showing standard deviation; graph is representative of 3 independent experiments. **b.** Confirmatory counter-screening of each compound with wild-type rSOSV. Huh7 cells were treated with serial dilutions of compound 1 h prior to infection with rSOSV at MOI 0.2. At 2 days post infection cells were fixed, and SOSV proteins stained and quantified using immunofluorescence microscopy. Relative fluorescence (red; total fluorescence normalized to mock-treated cells) in each well was determined, with cell viability assessed concurrently by determining ATP content (blue). Each point represents the mean of quadruplicate wells, with error bars showing standard deviation. Values stated are the 50% effective concentration (EC_50_), 50% cytotoxic concentration (CC_50_), and selectivity index (SI).

To confirm the inhibitory effect of 2′-dFC, 6-azauridine, and MPA in the context of authentic viral replication, we performed counter-screening with rSOSV/ZsG. Alongside these 3 compounds, we included 09167, an antagonist of the innate immune system that was shown to inhibit NiV and other paramyxoviruses in a similar screening assay [22,24]. Huh7 cells were treated with compound 1 h prior to infection with rSOSV/ZsG at an MOI of 0.2, with ZsG fluorescence read 48 hpi. All 3 compounds reduced ZsG expression in a dose-dependent manner, with EC_50_ values similar to those observed during the minigenome screen (Fig 4A). 2′-dFC (EC_50_ = 1.48 ± 0.22 μM) and 6-azauridine (EC_50_ = 7.99 ± 1.32 μM) potently inhibited ZsG fluorescence without cytotoxicity at the highest compound concentrations. While MPA had an EC_50_ value of 0.33 ± 0.04 μM, similar to that obtained in the minigenome screen, it again had a low SI of 5 due to its cytotoxicity at higher concentrations (Table 1). The 0.67 ± 0.05 μM EC_50_ value of 09167 was consistent with the sub-micromolar concentrations used to inhibit other paramyxoviruses, indicating that SOSV was similarly sensitive to this compound [24].

**Table 1:** Selected compounds’ 50% effective concentrations (EC_50_), 50% cytotoxic concentrations (CC_50_), and selectivity indices (SI) determined by minigenome and reporter virus screening assays.

Compound	Minigenome Screen		rSOSV/ZsG Screen		CC_50_	Compound Class
	EC_50_	SI	EC_50_	SI		
Ribavirin	5.91 ± 0.85	> 8	6.10 ± 0.09	> 8	> 50.0	Nucleoside analog
2′-deoxy-2′-fluorocytidine	1.31 ± 0.15	> 38	1.48 ± 0.22	> 33	> 50.0	Nucleoside analog
5-fluorouridine	0.29 ± 0.13	> 172	NT	NT	> 50.0	Nucleoside analog
Mycophenolic acid	± 0.06	7	0.33 ± 0.04	5	1.74 ± 0.42	IMPDH inhibitor
6-azauridine	5.17 ± 1.01	> 9	7.99 ± 1.32	> 6	> 50.0	Nucleoside analog
09167	NT	NT	0.67 ± 0.05	> 74	>50.0	Innate immune antagonist

NT, not tested; IMPDH, inosine monophosphate dehydrogenase. All values in μM ± standard deviation from at least 3 independent experiments.

**Fig 5:**
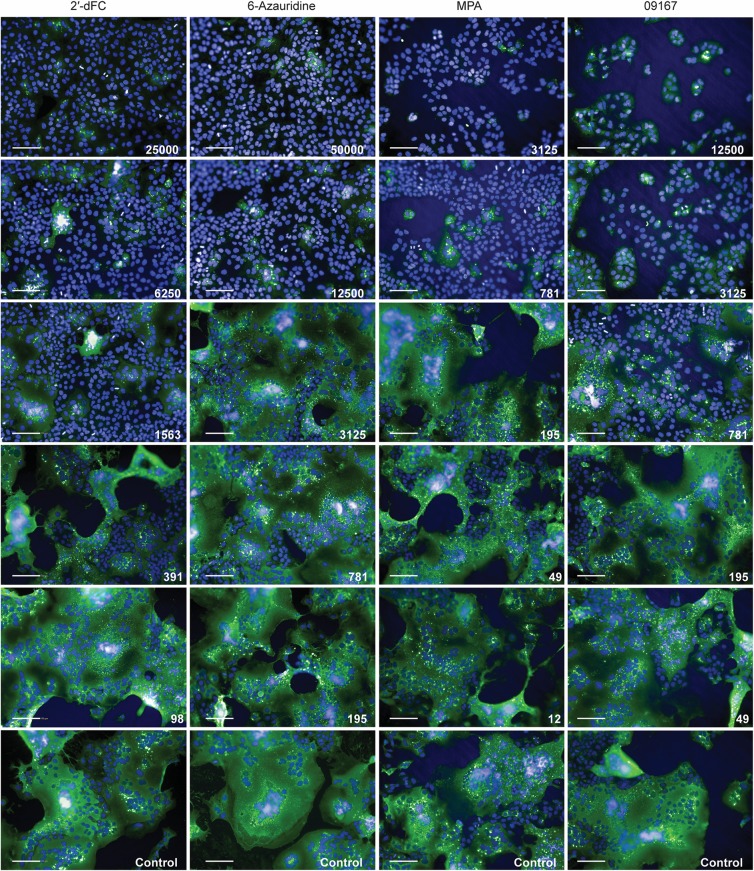
Immunofluorescent imaging of rSOSV infected Huh7 cells treated with selected antiviral compounds. Huh7 cells were treated with serial dilutions of either 2′-deoxy-2′-fluorocytidine (2′-dFC), 6-azauridine, mycophenolic acid (MPA), or 09167 1 h prior to infection with rSOSV at MOI 0.2. At 48 hpi, cells were fixed and SOSV proteins (green) and cell nuclei (blue) visualized by immunofluorescence. Concentration of compound is shown in white text on each representative image (mM); control cells were treated with DMSO only. Images taken using a 20 × objective. White bar represents 100 μm.

To ensure the validity of using the ZsG-expressing virus to accurately identify specific inhibitors, we investigated whether these compounds also inhibited the replication of rSOSV. Huh7 cells were treated with 2′-dFC, 6-azauridine, MPA, or 09167, and infected with rSOSV (MOI = 0.2) 1 h post treatment. At 48 hpi, cells were fixed and total SOSV protein production was visualized using rSOSV-specific and fluorescently labelled antibodies. Total fluorescence due to rSOSV protein staining per well (average of 16 analyzed fields per well) was normalized to rSOSV-infected, mock-treated cells, allowing determination of the relative reduction in virus replication due to the inhibitory effects of each compound. All compounds tested reduced rSOSV protein production in a dose-dependent manner, indicating inhibition of viral replication and growth (Fig 4B). Direct visualization of the compound-treated rSOSV infected cell monolayers clearly demonstrated the reduction in viral protein production, and also demonstrated the ability of rSOSV to form extensive syncytia in Huh7 cells at sub-optimal concentrations of compound (Fig 5). These results indicate that inhibition caused by the compounds is indeed due to a SOSV-specific mechanism, rather than one unique to the ZsG-expressing recombinant.

## Discussion

That SOSV, a previously unknown paramyxovirus vectored by bats, could cause severe disease in humans is not unexpected, given that bats are increasingly implicated as sources of novel zoonotic infections [3,25–27]. A recent study focusing on only 10% of bat species in relatively small geographic areas discovered evidence of 66 novel paramyxoviruses [28], so undiscovered pathogens with the capacity to threaten human health may yet be present in bat populations worldwide. With reverse genetics systems already developed for the highly pathogenic paramyxoviruses NiV and HeV, the establishment of SOSV reverse genetics based on similar design concepts ensures that any investigations into future outbreaks can be quickly initiated [20,21,29]. Furthermore, a common feature of paramyxoviruses appears to be that expression of reporter genes does not negatively affect the viral phenotype either in vitro or in vivo, increasing the usefulness of these systems [21,22,30,31]. The minigenome screening methods described here were successfully used to identify several compounds that inhibit SOSV replication. We were then able to confirm the antiviral activity of candidate compounds by using the reporter virus screen. While the minigenome screen is useful for identifying compounds that inhibit viral replication and transcription, the reporter virus screen can be utilized to identify other compounds, especially those that affect other viral life cycle processes, such as cell tropism, entry, budding, and transmission.

The antiviral activity of 6-azauridine and its derivatives toward several viruses has been reported, including for viruses closely related to SOSV, such as NiV, measles, and human parainfluenza virus 3 [22,32,33]. In a clinical setting, the derivative 6-azauridine triacetate has been used therapeutically to treat severe psoriasis for over 40 years, with minimal side effects observed at a dosage level of 125 mg/kg/day [34,35]. Encouragingly, research also shows that at that dose, plasma concentrations in human subjects reach 100 μM or greater, with successive doses over several days maintaining this level [36]. This is far in excess of the 5.17 μM EC_50_ for 6-azauridine reported here, so the potential usefulness in treating SOSV even at lower and less frequent doses should be further evaluated. 2′-dFC has been shown to potently inhibit several other negative strand viruses, including influenza and hepatitis C virus [37,38], although concerns over mitochondrial toxicity have limited the therapeutic use of 2′-dFC and its derivatives to date [39]. However, the potent antiviral activity of 2′-dFC makes worthwhile to investigate whether other 2′-deoxynucleoside analogs have similar antiviral activity against SOSV in the absence of toxicity.

The antiviral activity demonstrated by ribavirin was expected, and has been reported previously for NiV [22,40,41]. Ribavirin has also been used to treat human patients during a non-randomized, unblinded limited clinical trial during an NiV outbreak in Malaysia in 1998–1999; mortality was reduced by 37% in the treated group [42]. However, its effectiveness when used in NiV animal models is less clear [40,43,44]. A similar discrepancy between in vivo and in vitro potency of these compounds may exist for SOSV, although this will remain unclear until a suitable SOSV animal model is developed. Promisingly, the limiting factor to henipavirus treatment effectiveness appears to be the compound’s ability to cross the blood-brain barrier (BBB). Ribavirin treatment in a non-human primate model of HeV infection reduced viral loads to levels low enough to prevent development of the respiratory signs of infection, but not to prevent spread to the central nervous system [44]. As ribavirin cannot cross the BBB, the disease then manifested as neurological signs. However, no neurological symptoms were reported from the one SOSV case to date, so nucleoside analogs such as ribavirin, 6-azauridine, and 2′-dFC may be more effective treatment options for this disease.

In conclusion, we have developed a minigenome assay to investigate SOSV replication and transcriptional processes, as well as a rescue system to recover infectious recombinant virus. We used this system to rescue a recombinant SOSV capable of expressing a fluorescent reporter in infected cells with no loss of fitness compared to parental virus. A high-throughput screen using both systems identified 3 compounds that inhibited SOSV replication with acceptable SI values, providing information that may help direct therapeutic options for any future cases of SOSV. Finally, as well as being a powerful tool for analyzing mechanisms of SOSV pathogenesis, the minigenome and reporter systems may help us better understand the potential public health threats posed by bat-transmitted paramyxoviruses, and provide strategies on best combating these emerging threats.

## Supporting information

S1 Fig Plasmids use in the SOSV minigenome assay. The SOSV minigenome (mg) segment, containing the SOSV leader sequence and gene start for the nucleoprotein ORF, the ZsGreen1 ORF (ZsG), the polymerase ORF gene end, and the SOSV trailer sequence (Genbank #MG880223). Expression is controlled RNA-polymerase I promoter (pol I prom) and a RNA-polymerase I terminator sequences (pol I term).
(TIF)

S2 Fig Plasmids used in the rescue of rSOSV (pSOSV FL) and rSOSV/ZsG (pSOSV/ZsG FL). Red arrows represent full-length viral genome, blue arrows represent viral protein open reading frames: NP–nucleoprotein; P–phosphoprotein; V–V protein; M–matrix protein; F–fusion protein; HN–hemagglutinin-neuraminidase; L–polymerase. Green arrow represents ZsGreen1 (ZsG) open reading frame. T7 prom–T7 polymerase promoter; HDV Rz–Hepatitis D virus ribozyme; T7term–T7 polymerase terminator; P2A –nucleotide sequence encoding the self-cleaving amino acid motif from porcine teschovirus 1. Sequences available at: pSOSV FL, Genbank #MG880224; pSOSV/ZsG FL, Genbank #MG880225).
(TIF)

S1 Table Screening a panel of selected compounds for inhibition of SOSV minigenome in Huh7 cells. Compounds were screened for activity at concentrations of 5000, 500, and 50 nM, with cell viability determined concurrently by assessing ATP content.
(DOCX)
