# Supplementary figures and images for: Development of a reverse genetics system for Sosuga virus allows rapid screening of antiviral compounds
Source: PLoS Negl Trop Dis. 2018 Mar 9;12(3):e0006326. doi: 10.1371/journal.pntd.0006326 (PMC5862516; doi:10.1371/journal.pntd.0006326)

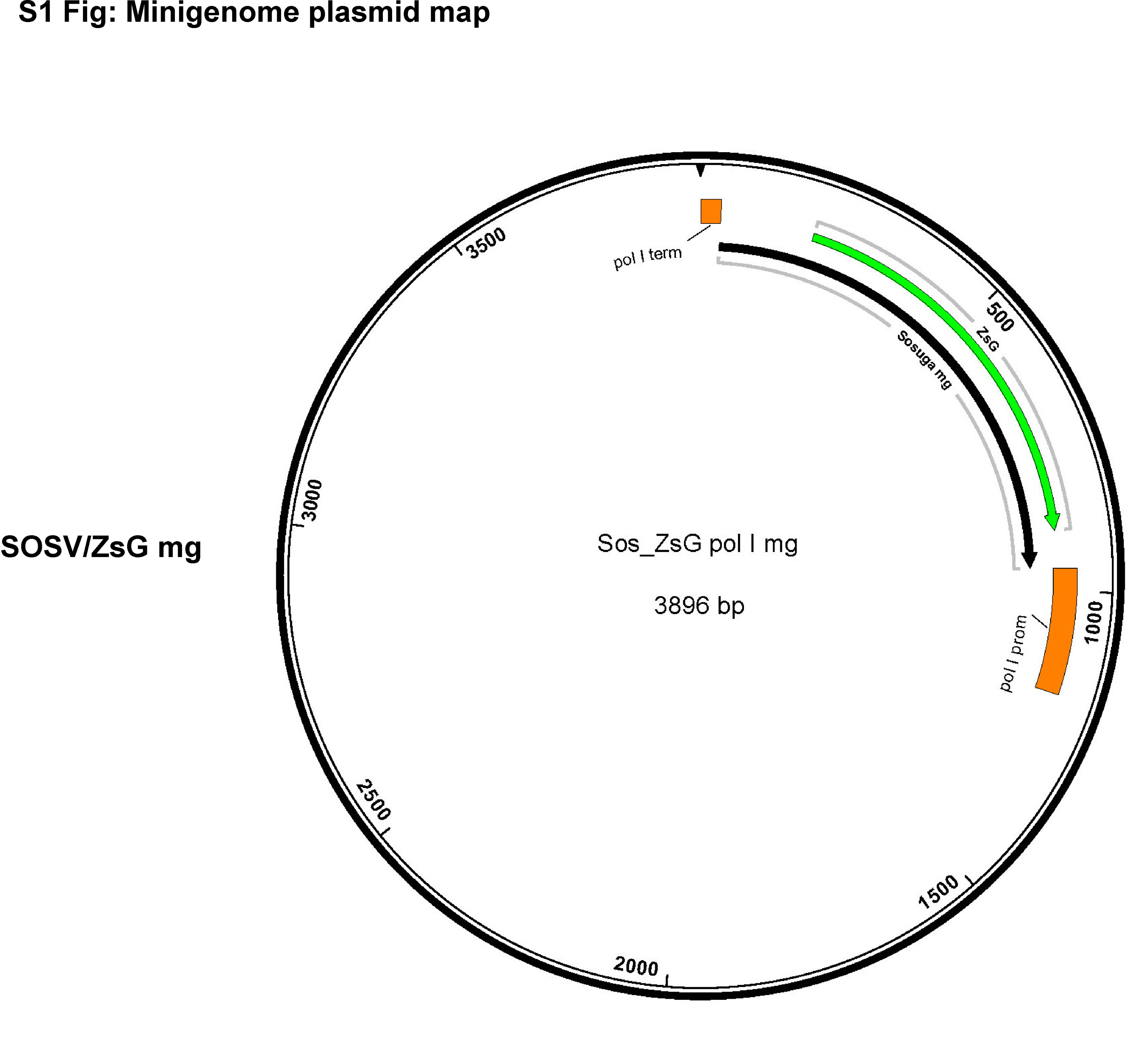

Supplement: S1 Fig — The SOSV minigenome (mg) segment, containing the SOSV leader sequence and gene start for the nucleoprotein ORF, the ZsGreen1 ORF (ZsG), the polymerase ORF gene end, and the SOSV trailer sequence (Genbank #MG880223). Expression is controlled RNA-polymerase I promoter (pol I prom) and a RNA-polymerase I terminator sequences (pol I term). (TIF) [file pntd.0006326.s001.tif]

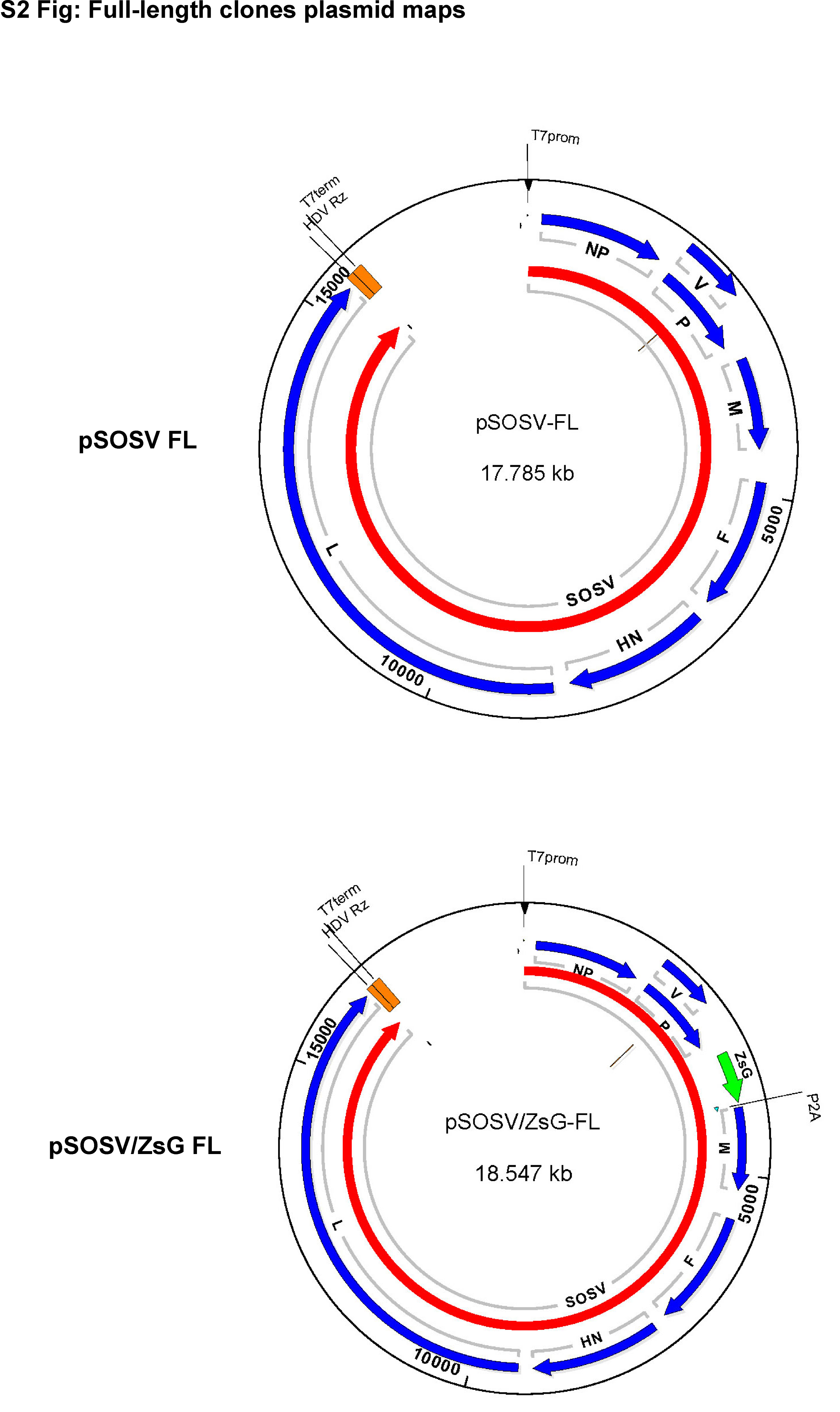

Supplement: S2 Fig — Red arrows represent full-length viral genome, blue arrows represent viral protein open reading frames: NP–nucleoprotein; P–phosphoprotein; V–V protein; M–matrix protein; F–fusion protein; HN–hemagglutinin-neuraminidase; L–polymerase. Green arrow represents ZsGreen1 (ZsG) open reading frame. T7 prom–T7 polymerase promoter; HDV Rz–Hepatitis D virus ribozyme; T7term–T7 polymerase terminator; P2A –nucleotide sequence encoding the self-cleaving amino acid motif from porcine teschovirus 1. Sequences available at: pSOSV FL, Genbank #MG880224; pSOSV/ZsG FL, Genbank #MG880225). (TIF) [file pntd.0006326.s002.tif]
