# Supplementary material for: Development of a reverse genetics system for Sosuga virus allows rapid screening of antiviral compounds
Source: PLoS Negl Trop Dis. 2018 Mar 9;12(3):e0006326. doi: 10.1371/journal.pntd.0006326 (PMC5862516; doi:10.1371/journal.pntd.0006326)
Supplement: S1 Table — Compounds were screened for activity at concentrations of 5000, 500, and 50 nM, with cell viability determined concurrently by assessing ATP content. (DOCX) [file pntd.0006326.s003.docx]

**S1 Table:** Screening a panel of selected compounds for inhibition of SOSV minigenome in Huh7 cells.

| **Compound** | **Minigenome activity**  **(Relative ZsG fluorescence)** | | | | | | | | | **Cell viability**  **(Relative ATP content)** | | | | | | | | | **Compound description** |
| --- | --- | --- | --- | --- | --- | --- | --- | --- | --- | --- | --- | --- | --- | --- | --- | --- | --- | --- | --- |
|  |  | | | | | | | | |  | | | | | | | | |  |
|  | **5000 nM** | | | **500 nM** | | | **50 nM** | | | **5000 nM** | | | **500 nM** | | | **50 nM** | | |  |
| PSI-7976 | 0.64 | ± | 0.05 | 0.94 | ± | 0.11 | 0.88 | ± | 0.06 | 0.94 | ± | 0.10 | 0.87 | ± | 0.04 | 0.89 | ± | 0.01 | Inhibitor of HCV replication |
| PSI-7977 | 0.89 | ± | 0.10 | 1.02 | ± | 0.09 | 1.02 | ± | 0.01 | 1.05 | ± | 0.10 | 0.90 | ± | 0.02 | 0.92 | ± | 0.00 | HCV RdRp inhibitor |
| R-1479 | 0.73 | ± | 0.09 | 0.84 | ± | 0.05 | 0.91 | ± | 0.05 | 0.93 | ± | 0.08 | 0.86 | ± | 0.00 | 0.89 | ± | 0.01 | Nucleoside analog |
| R-7128 | 0.99 | ± | 0.12 | 0.87 | ± | 0.10 | 0.84 | ± | 0.08 | 0.97 | ± | 0.06 | 0.87 | ± | 0.02 | 0.89 | ± | 0.00 | Nucleoside analog |
| Balapiravir (R1626) | 0.90 | ± | 0.11 | 0.93 | ± | 0.27 | 1.05 | ± | 0.13 | 0.98 | ± | 0.09 | 0.85 | ± | 0.01 | 0.89 | ± | 0.02 | Prodrug of R-1479 |
| PSI-6130 | 0.91 | ± | 0.04 | 0.85 | ± | 0.06 | 0.84 | ± | 0.08 | 1.00 | ± | 0.07 | 0.90 | ± | 0.02 | 0.91 | ± | 0.02 | HCV polymerase |
| PSI-6206 | 1.14 | ± | 0.15 | 1.14 | ± | 0.28 | 0.87 | ± | 0.05 | 1.01 | ± | 0.05 | 0.88 | ± | 0.02 | 0.88 | ± | 0.01 | HCV polymerase |
| ABT-333 | 1.05 | ± | 0.14 | 1.08 | ± | 0.24 | 1.27 | ± | 0.02 | 1.01 | ± | 0.09 | 0.96 | ± | 0.00 | 0.98 | ± | 0.03 | HCV non-nucleoside inhibitor |
| NITD008 | 1.13 | ± | 0.08 | 1.07 | ± | 0.15 | 1.35 | ± | 0.07 | 1.03 | ± | 0.03 | 0.95 | ± | 0.02 | 0.98 | ± | 0.02 | Nucleoside analog |
| Azithromycin | 1.11 | ± | 0.15 | 1.06 | ± | 0.11 | 1.02 | ± | 0.14 | 1.15 | ± | 0.07 | 1.00 | ± | 0.01 | 0.99 | ± | 0.01 | Protein synthesis inhibitor |
| Nucleoside analog 1 | 1.15 | ± | 0.29 | 0.93 | ± | 0.10 | 1.02 | ± | 0.15 | 1.15 | ± | 0.06 | 1.01 | ± | 0.01 | 0.99 | ± | 0.01 | Nucleoside analog |
| Nucleoside analog 2 | 1.33 | ± | 0.28 | 0.96 | ± | 0.11 | 1.30 | ± | 0.30 | 1.13 | ± | 0.03 | 1.02 | ± | 0.02 | 1.00 | ± | 0.02 | Nucleoside analog |
| R09187 | 1.11 | ± | 0.26 | 1.05 | ± | 0.12 | 1.04 | ± | 0.09 | 1.06 | ± | 0.01 | 1.02 | ± | 0.01 | 1.02 | ± | 0.00 | Virus replication inhibitor |
| Tenofovir | 1.07 | ± | 0.22 | 1.06 | ± | 0.12 | 1.10 | ± | 0.02 | 1.13 | ± | 0.07 | 1.02 | ± | 0.02 | 0.99 | ± | 0.02 | NARTI, HIV, HBV |
| 2′-Deoxy-2′-fluorocytidine | 0.13 | ± | 0.07 | 0.91 | ± | 0.14 | 1.41 | ± | 0.13 | 1.07 | ± | 0.06 | 1.02 | ± | 0.03 | 1.01 | ± | 0.01 | Nucleoside analog |
| 2-Chloroadenosine | 1.45 | ± | 0.25 | 1.43 | ± | 0.26 | 1.48 | ± | 0.01 | 1.08 | ± | 0.01 | 1.03 | ± | 0.00 | 1.02 | ± | 0.01 | Nucleoside analog |
| 2′-C-Methylcytidine | 1.14 | ± | 0.18 | 1.04 | ± | 0.08 | 1.24 | ± | 0.09 | 1.10 | ± | 0.01 | 1.02 | ± | 0.00 | 1.00 | ± | 0.00 | Nucleoside analog |
| 2′-C-Methyluridine | 1.25 | ± | 0.14 | 1.01 | ± | 0.16 | 1.32 | ± | 0.17 | 1.12 | ± | 0.04 | 1.02 | ± | 0.02 | 1.01 | ± | 0.01 | Nucleoside analog |
| 2′-O-Methylcytidine | 1.19 | ± | 0.13 | 1.04 | ± | 0.11 | 1.29 | ± | 0.14 | 1.08 | ± | 0.00 | 1.02 | ± | 0.02 | 1.01 | ± | 0.00 | Nucleoside analog |
| 2′-O-Methyluridine | 1.42 | ± | 0.25 | 1.04 | ± | 0.15 | 1.50 | ± | 0.04 | 1.08 | ± | 0.00 | 1.03 | ± | 0.00 | 1.01 | ± | 0.00 | Nucleoside analog |
| Cordycepin | 1.01 | ± | 0.13 | 1.04 | ± | 0.13 | 1.28 | ± | 0.12 | 1.06 | ± | 0.01 | 1.01 | ± | 0.00 | 0.98 | ± | 0.01 | 3-deoxyadenosine |
| 5-Azacytidine | 1.08 | ± | 0.16 | 1.11 | ± | 0.23 | 1.46 | ± | 0.06 | 1.12 | ± | 0.10 | 1.00 | ± | 0.02 | 1.00 | ± | 0.02 | Nucleoside analog |
| 5-Bromouridine | 1.22 | ± | 0.10 | 0.92 | ± | 0.11 | 0.97 | ± | 0.14 | 1.17 | ± | 0.09 | 1.05 | ± | 0.02 | 1.01 | ± | 0.00 | Nucleoside analog |
| 5-Chlorouridine | 1.46 | ± | 0.21 | 1.25 | ± | 0.13 | 1.19 | ± | 0.07 | 1.13 | ± | 0.04 | 1.04 | ± | 0.00 | 1.01 | ± | 0.02 | Nucleoside analog |
| 5-Methylcytidine | 1.25 | ± | 0.21 | 1.10 | ± | 0.19 | 1.24 | ± | 0.10 | 1.07 | ± | 0.02 | 1.02 | ± | 0.01 | 1.01 | ± | 0.02 | Nucleoside analog |
| 6-Aza-2-thiouridine | 1.10 | ± | 0.29 | 0.95 | ± | 0.21 | 1.28 | ± | 0.08 | 1.07 | ± | 0.02 | 1.04 | ± | 0.03 | 1.03 | ± | 0.01 | Nucleoside analog |
| 5-Azidouridine | 1.07 | ± | 0.13 | 1.04 | ± | 0.26 | 1.14 | ± | 0.04 | 1.08 | ± | 0.01 | 1.02 | ± | 0.04 | 1.00 | ± | 0.02 | Nucleoside analog |
| Stavudine | 1.15 | ± | 0.08 | 1.04 | ± | 0.12 | 1.26 | ± | 0.10 | 1.05 | ± | 0.00 | 1.02 | ± | 0.00 | 1.02 | ± | 0.01 | NARTI, HIV |
| Zidovudine | 1.13 | ± | 0.15 | 0.93 | ± | 0.13 | 1.23 | ± | 0.16 | 0.98 | ± | 0.04 | 0.98 | ± | 0.01 | 0.96 | ± | 0.01 | NARTI, HIV |
| Lamuvudine | 1.27 | ± | 0.24 | 1.13 | ± | 0.08 | 1.38 | ± | 0.07 | 1.14 | ± | 0.10 | 1.01 | ± | 0.00 | 0.99 | ± | 0.02 | NARTI, HIV |
| 5-Fluorouridine | 0.10 | ± | 0.02 | 0.25 | ± | 0.03 | 1.12 | ± | 0.21 | 1.09 | ± | 0.09 | 0.97 | ± | 0.00 | 1.02 | ± | 0.01 | Nucleoside analog |
| 7-DMA | 1.06 | ± | 0.08 | 1.02 | ± | 0.19 | 1.26 | ± | 0.14 | 1.13 | ± | 0.07 | 1.04 | ± | 0.00 | 1.00 | ± | 0.00 | Nucleoside analog |
| 6-Azauridine | 0.46 | ± | 0.09 | 1.03 | ± | 0.07 | 1.20 | ± | 0.04 | 1.21 | ± | 0.05 | 1.04 | ± | 0.00 | 1.03 | ± | 0.02 | Nucleoside analog |
| 8-Azaadenosine | 0.02 | ± | 0.01 | 0.85 | ± | 0.12 | 1.46 | ± | 0.12 | 0.51 | ± | 0.00 | 1.00 | ± | 0.03 | 1.00 | ± | 0.00 | Nucleoside analog |
| 8-Azidoadenosine | 0.01 | ± | 0.01 | 1.22 | ± | 0.05 | 1.20 | ± | 0.13 | 0.30 | ± | 0.01 | 0.91 | ± | 0.02 | 1.00 | ± | 0.00 | Nucleoside analog |
| 3-Aeazauridine | 1.17 | ± | 0.10 | 1.10 | ± | 0.01 | 1.15 | ± | 0.14 | 1.04 | ± | 0.03 | 1.03 | ± | 0.01 | 1.01 | ± | 0.00 | Nucleoside analog |
| BCX-4430 | 1.18 | ± | 0.26 | 1.05 | ± | 0.12 | 1.33 | ± | 0.24 | 1.02 | ± | 0.04 | 1.03 | ± | 0.03 | 1.01 | ± | 0.01 | RdRp inhibitor |
| T-705 | 1.05 | ± | 0.03 | 1.17 | ± | 0.21 | 1.11 | ± | 0.08 | 1.03 | ± | 0.03 | 1.02 | ± | 0.00 | 1.01 | ± | 0.00 | Akt/PKB inhibitor |
| Mycophenolic acid (MPA) | 0.01 | ± | 0.01 | 0.14 | ± | 0.03 | 0.89 | ± | 0.14 | 0.39 | ± | 0.02 | 0.92 | ± | 0.04 | 1.07 | ± | 0.06 | IMPDH inhibitor |
| Ribavirin | 0.78 | ± | 0.15 | 1.13 | ± | 0.19 | 1.36 | ± | 0.13 | 1.17 | ± | 0.02 | 1.05 | ± | 0.05 | 0.99 | ± | 0.08 | Nucleoside analog |

RdRp, RNA-dependent RNA polymerase; HCV, hepatitis C virus; NARTI, nucleoside analog reverse transcriptase inhibitor; HIV, human immunodeficiency virus; HBV, hepatitis B virus; IMPDH, inosine monophosphate dehydrogenase. Experiments were performed in triplicate, with all values relative to mock-treated controls ± standard deviation.
